# Male-Produced (−)-δ-Heptalactone, Pheromone of Fruit Fly *Rhagoletis batava* (Diptera: Tephritidae), a Sea Buckthorn Berries Pest

**DOI:** 10.3390/insects11020138

**Published:** 2020-02-23

**Authors:** Vincas Būda, Laima Blažytė-Čereškienė, Sandra Radžiutė, Violeta Apšegaitė, Patrick Stamm, Stefan Schulz, Dominykas Aleknavičius, Raimondas Mozūraitis

**Affiliations:** 1 Laboratory of Chemical Ecology, Nature Research Centre, Akademijos 2, LT-08412 Vilnius, Lithuania; vincas.buda@gamtc.lt (V.B.); laima.blazyte@gamtc.lt (L.B.-Č.); sandra.radziute@gamtc.lt (S.R.); violeta.apsegaite@gamtc.lt (V.A.); dominykas.aleknavicius@gamtc.lt (D.A.); 2 Institute of Organic Chemistry, Technische Universität Braunschweig Hagering 30, 38106 Braunschweig, Germany; p.stamm@tu-braunschweig.de (P.S.);

**Keywords:** *Rhagoletis batava*, pheromone identification, synthesis, bioassay, field trapping, SPME, GC-EAD, GC-MS

## Abstract

The plantation area of sea buckthorn (*Hippophae rhamnoides* L.) is expanding in many European countries due to increasing demand for berries, thus creating suitable conditions for the rapid expansion of the fruit fly *Rhagoletis batava*, a pest of economic importance. To decrease insecticide use, effective means for pest population monitoring are required, including the use of pheromones. Male fruit flies emit (-)-δ-heptalactone as revealed by gas chromatography-mass spectrometry analyses of samples obtained using headspace methods. The two enantiomers of δ-heptalactone were synthesized using enantioselective synthesis. A gas chromatography-electroantennographic detection analysis of both stereoisomers revealed that only (-)-δ-heptalactone elicited electrophysiological responses, whereas no signal was registered to (+)-δ-heptalactone in fruit flies of either sex. In the field assay, traps baited with (-)-δ-heptalactone caught significantly more fruit flies compared with the unbaited traps. Our results are the first to demonstrate the efficacy of (-)-δ-heptalactone as a bait for trapping *R. batava*. As a behaviorally attractive compound to *R. batava* fruit flies of both sexes, (-)-δ-heptalactone is attributed to aggregation pheromones. This is the first report of an aggregation pheromone within the genus *Rhagoletis*.

## 1. Introduction

*Rhagoletis batava* Hering (Diptera: Tephritidae) fruit flies are pests of economic importance that cause damage to sea buckthorn (*Hippophae rhamnoides* L. Rosales: Elaeagnaceae) berries. After a female fruit fly lays eggs on a sea buckthorn berry, the hatched larvae enter a berry and feed inside; eventually, infested berries change color from natural yellow to black and lose their commercial and nutritional value [1,2]. As damaged berries remain stuck to branches alongside undamaged berries, harvesting both decreases the value of the total yield. Sea buckthorn is cultivated in European countries due to the multiple ecological and medicinal benefits of sea buckthorn [3,4]. The cultivar was introduced from Asia and, since introduction, the plantation area has increased significantly, thus providing conditions conducive to spreading natural pests such as *R. batava*.

Demand for sea buckthorn berries for food, medical purposes and cosmetics is increasing [3,5,6,7,8] and supply has not been able to satisfy this demand [9,10]. The production of organic sea buckthorn is a special demand. However, to date, no environmentally friendly means of pest control are available for this species; thus, insecticides are widely used for yield protection. Current tools available for monitoring *R. batava* populations are ineffective. Yellow sticky traps are still recommended and used [11] and the use of nonspecific attractant has also been recently reported [12]. Pheromone identification could fill this gap and be applied for pest population monitoring and potentially contribute to environmentally friendly pest control. The latter application seems especially promising, as males mainly produce pheromones that affect the behavior of females in a few species of genus *Rhagoletis* fruit flies [13,14].

The pheromone mediating attraction of *Rhagoletis* flies has been identified for walnut husk fly, *Rhagoletis completa* Cresson (Diptera: Tephritidae). Two lactones, namely δ-hexalactone and δ-heptalactone at the ratio 4:6, were released exclusively by males and showed significant attractiveness under field experiments [15]. Unfortunately, the sex of the specimens caught in the traps was not determined; hence, information is insufficient to determine whether one or both sexes responded to the lactones. Raptopoulos et al. [16] proposed that pheromone produced by *Rhagoletis cerasi* (Diptera: Tephritidae) males is a multicomponent blend; however, the chemical composition of volatiles remains putative. Up to date, pheromones of *R. batava* have not been previously studied.

The purpose of this study was to isolate and identify pheromones in *R. batava*, to synthesize the compound(s) and to evaluate the biological activity of synthetics both on olfactory receptors and on a behavioral level under laboratory as well as field conditions.

## 2. Materials and Methods 

### 2.1. Insects

For the electrophysiological recordings, sea buckthorn fruit flies, *Rhagoletis batava*, were collected as puparia from the soil under sea buckthorn shrubs in May 2019 at an organic sea buckthorn *Hippophae rhamnoides* L. plantation (24 ha area, global positioning system (GPS) coordinates: 55°15′12.179″ N, 25°26′23.049″ E) in Stacijava village, Molėtai district, Lithuania. Each puparium was placed in an individual 14 mL glass vial containing wet 3 cm^2^ filter paper and corked by foam stoppers. Vials were placed in a Fitotron climate chamber (Weiss Gallenkamp, Loughborough, UK), under 20–24 °C, for a 16:8 light:dark photoperiod and 65–75% relative humidity. Twice per week, 2–3 drops of water were added to the filter paper to maintain high humidity inside the vial. Emerged adults were maintained in the same vials in a walk-in climate room at 18–20 °C, natural daylight photoperiod, 50–60% relative humidity and fed on 10% sugar solution in water. 

The flies were sexed by visually examining the abdomen tip; the presence of an ovipositor indicated that the specimen was a female. The sexed flies were maintained in individual vials under the same conditions described above. 

### 2.2. Sampling of R. batava-Produced Volatiles

Solid phase micro-extraction (SPME) was used to sample the headspace of the fruit flies. Before each collection, the SPME fibers coated with polydimethylsiloxane-divinylbenzene polymer (DVB/PDMS, 65 µm coating layer thickness, Supelco, Bellefonte, PA, USA) were routinely purified at 240 °C, lasting for about 10 min in a GC injector. For SPME, between 8 and 20 adult fruit flies (4–8 days old, either males or females) were placed in a 50 mL glass vial covered in aluminum foil and exposed to the fiber for 120 min. Once sampling was finished, the fiber was transferred to the injection port, either of the coupled gas chromatography-electroantennogram detection (GC-EAD) or gas chromatography-mass spectrometry (GC-MS) system, operating under the conditions described below. The thermal desorption duration of volatiles from the fiber was 2 min. Headspace SPME samples were collected from females and males of *R. batava* and analyzed by GC-EAD using antennae of both sexes. 

### 2.3. GC-EAD Detection

GC-EAD (gas chromatograph Clarus 500, PerkinElmer, Waltham, MA, USA; EAD, Ockenfels Syntech GmbH, Buchenbach, Germany) was used to identify EAD-active compound(s) in the headspace of *R. batava* flies and to test EAD activity of synthetic compounds. 

The GC effluent was divided using a splitter into two equal parts, allowing simultaneous flame ionization and EAD detection of the separated volatile compounds. One part of the column effluent was directed to the flame ionization detector (FID). A nitrogen make-up gas at a flow rate of 5 mL/min was used to enhance FID performance. Another part of the effluent was introduced into a purified and humidified air stream flowing at 0.5 m/s through a glass tube over antenna preparation. The flies used in GC-EAD analyses were not chilled or anesthetized prior to use. Glass capillary electrodes were filled with 0.9% NaCl saline (Ilsanta, Vilnius, Lithuania) and grounded via a silver wire. An indifferent electrode was inserted into the severed head of the fly. The recording electrode, connected to a high-impedance DC amplifier (IDAC-4, Ockenfels Syntech GmbH, Buchenbach, Germany) with automatic baseline drift compensation was brought into contact with the distal end of the fly antenna. The antennal and the FID signals were recorded simultaneously, stored and analyzed using GcEad V. 4.4 software (Synthech, Ockenfels Syntech GmbH, Buchenbach, Germany). The male and female antennae responses to male odors and to synthetic (+)- and (-)-δ-heptalactones, as well as male antenna responses to female odors were tested. Flies from 4 to 7 days old were used in the tests. Each EAD test was replicated three times and each antenna used was from a different fly.

For headspace analysis, the GC was equipped with a DB-Wax capillary column (30 m × 0.25 mm × 0.25 µm; Agilent Technologies, Santa Clara, CA, USA). The injector and the detector temperatures were set to 240 °C. The oven temperature was maintained isothermally at 40 °C for 1 min; afterward, it was raised to 240 °C at a rate of 10 °C/min, then maintained isothermally for 13 min. Hydrogen, at a flow rate 1.5 mL/min, was used as a carrier gas.

After the identification of EAD active compounds and revealing the potential presence of enantiomers, a Rt^®^-bDEXsm column (30 m × 0.25 mm × 0.25 µm, Restek Corporation, Bellefonte, PA, USA) was used instead of the previous column. The stationary chiral phase of the column was composed of 2,3-di-O-methyl-6-O-tert-butyl dimethylsilyl β cyclodextrin, added into 14% cyanopropylphenyl/86% dimethyl polysiloxane. Cyclodextrin-based GC stationary phases provide excellent separation for a wide range of chiral compounds and are the most widely used [17]. In this case, the injector and the detector temperatures were set to 230 °C. The oven initial temperature was 90 °C, then programmed to 200 °C at a rate of 3 °C/min.

### 2.4. GC-MS Analysis

A Shimadzu GC-2010 gas chromatograph coupled with a Shimadzu MS-QP 2010 Plus mass selective detector (Shimadzu, Kyoto, Japan) was used. The EAD active compound present in the *R. batava* flies’ headspace was analyzed using the GC equipped with a Stabil-Wax column (30 m × 0.25 mm × 0.25 µm, Restek Corporation, Bellefonte, PA, USA). For analysis of synthetic enantiomers as well as those present in the headspace, we used a Rt^®^-bDEXsm column (30 m × 0.25 mm × 0.25 µm, Restek Corporation, Bellefonte, PA, USA). The GC was operated under the same conditions as described in in Section 2.3, except that helium was used as the carrier gas, at a flow rate 1.5 mL/min. Electron ionization spectra were acquired at an electron energy of 70 eV and the interface and ion source temperatures were held isothermal at 250 °C. The headspace EAD active compound was identified by comparing its mass spectral data with those available from the library of National Institute of Standards and Technology (NIST, version 2.0, National Institute of Standards and Technology, Gaithersburg, MD, USA) and comparing the retention index with that of the synthetic standard compound. The retention index [18] of the δ-heptalactone was calculated using the retention data from C_8_–C_28_ n-alkanes solution (Sigma-Aldrich Sweden) obtained under the same chromatographic parameters as those used for the analysis of the headspace samples.

### 2.5. Synthesis

**Figure 1:**
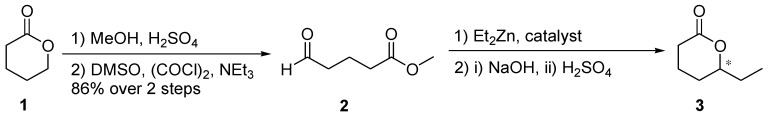
Synthesis of (+)- and (−)-δ-heptalactones.

Methanolysis of tetrahydro-2-*H*-pyran-2-one (1), followed by Swern oxidation according to Reid et al. (2018) [19], yielded methyl 5-oxo-pentanoate(2) which was transformed to 6-ethyltetrahydro-2*H*-pyran-2-one (δ-heptalactone) (3) in both enantiomeric forms by *N*,*N*-dibutylnorephidrine catalysed alkylation with diethylzinc followed to lactonization according to Soai et al. (1988) [20] (Figure 1). The enantiomeric excess was determined by chiral GC (hydrodex β 6TBDM column, initial temperature 85 °C hold for 30 min, afterwards raised to 95 °C at a rate of 1 °C/min and then raised to 230 °C at a rate of 30 °C/min) (Table 1).

R_f_ = 0.30 (Et_2_O/pentane 3:1);

^1^H NMR (CDCl_3_, 400 MHz) *δ* 4.26–4.19 (1H, m, CH), 2.63–2.54 (1H, m, CH*H*), 2.50–2.36 (1H, m, C*H*H), 1.97–1.47 (6H, m, 3 CH_2_), 1.00 (3H, t, *J* = 7.5 Hz, CH_3_);

^13^C NMR (CDCl_3_, 100 MHz) *δ* 171.9 (C), 81.8 (CH), 29.4 (CH_2_), 28.7 (CH_2_), 27.2 (CH_2_), 18.4 (CH_2_), 9.3 (CH_3_);

EIMS *m*/*z* 128 (5, [M]^+^), 100 (25), 99 (100), 71 (72), 70 (33), 57 (15), 56 (38), 55 (43), 43 (36), 42 (64), 41 (43), 39 (23).

(*R*)-(+)-(**3**):

${\left[\alpha \right]}_{D}^{20}$ = +40.4 (*c* 1.61, THF);

*ee* = 84%.

(*S*)-(–)-(**3**):

${\left[\alpha \right]}_{D}^{20}$ = −41.2 (*c* 1.62, THF);

*ee* = 86%.

### 2.6. EAG Dose-Response

Using the same electrophysiological recording setup and the same antennal preparation technique, electroantennogram (EAG) dose-responses of male and female *R. batava* to the synthetic δ-heptalactone were recorded. 

The compound was tested at doses of 10^−5^, 10^−4^, 10^−3^ and 10^−2^ μL following the application of 10 μL solution on a piece of filtered paper (5 × 45 mm) (Whatman^®^1, Kent, UK) and placed to a Pasteur pipette (Gmbh + Co Kg, Wertheim, Germany). Hexane solutions were prepared via the 10-step dilution of the previous solution. The four doses were tested in ascending order. A solvent blank (10 μL of hexane after evaporation) was tested as a control stimulus both at the beginning and the end of stimulation. The peak voltage amplitude was recorded during the puff delivery of each stimulus as an antenna response. Each stimulation was followed by at least a 1 min purge period of filtered air to ensure the recovery of the antennal receptors. Each EAD test was replicated five times and each antenna used was from a different fly. The EAG response (R) to the EAD-active compound dose was calculated according to the formula R = RA − (RC_1_ + RC_2_)/2, where RA is the EAG response to the EAD active compound and RC1 and RC2 are EAG responses to the first and the second control stimuli, respectively.

### 2.7. Field Assay

**Figure 2:**
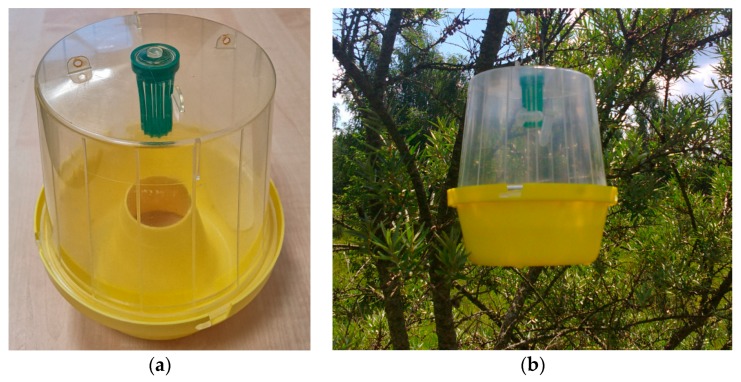
The McPhail trap: (**a**) empty trap and (**b**) trap fixed on the branch of sea buckthorn (*Hippophae rhamnoides*).

An organic sea buckthorn plantation in Stacijava village, Molėtai district, Lithuania was chosen for the field assay. McPhail (Pherobank, Wijk bij Duurstede, The Netherlands) traps, which are designed for catching flies, were used. Flies are attracted by the pheromone loaded in a dispenser and placed in a holder inside a trap. The attracted flies fly into the trap through an inverted funnel at the base of the trap, remained attracted by the pheromone and the daylight and do not find their way out. After a while, the flies fall into a soapy water solution at the base of the trap and drown (Figure 2).

Red rubber dispensers with inner diameters of 7.1 mm (5 × 11 mm), Pherobank, Wijk bij Duurstede, the Netherlands) were loaded with 25 µL of (-)-δ-heptalactone dissolved in 50 µL hexane. Control dispensers were loaded with the same amount of the solvent. For 20 min, the solvent was allowed to evaporate. Following evaporation, unloaded dispenser was inserted in each loaded dispenser to reduce emission of the test compound. Dispensers were placed into traps and, after a three-day period, were placed in the plantation at a distance of at least 4 m between the test and control traps and at a distance between such couples of approximately 50 m. Three pheromone-baited and three control traps were used. The field assay was conducted from 30 July to 20 August 2019, during flight period of *R. batava* flies [21]. The traps were inspected twice a week and the flies caught were taken to the laboratory for sex determination.

### 2.8. Statistical Analysis

EAG amplitudes of *R. batava* antennae of both sexes were compared using a Mann-Whitney U test at the level of significance α = 0.05. Field assay data of *R. batava* daily catches were transformed (x + 0.5)^1/2^ and a general linear model (GLM) was used to evaluate the sex (male or female) and treatment type (pheromone and control) effect on the attraction of flies [22]. The statistical analysis was performed using Statistica 6.0 and the GLM test was carried out by Statistica 13.5 software (StatSoft, Inc., Tulsa, OK, USA).

## 3. Results

### 3.1. EAD Active Compound

**Figure 3:**
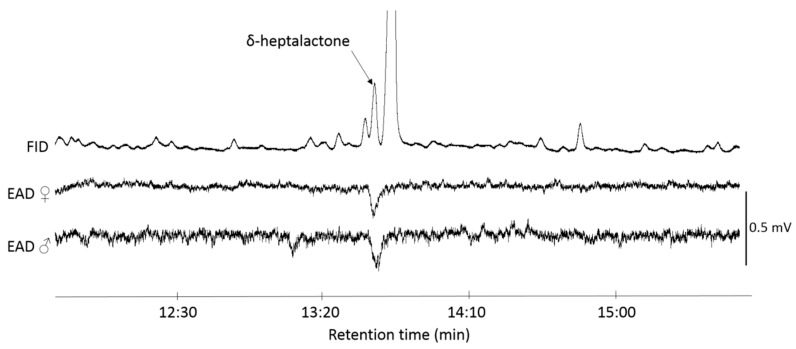
Gas chromatography-electroantennogram detection (GC-EAD) response of male and female *Rhagoletis batava* to headspace volatiles of *R. batava* males. FID, flame ionization detector; EAD, electroantennographic detector; DB-Wax capillary column (30 m × 0.25 mm × 0.25 µm; Agilent Technologies, Santa Clara, CA, USA); each EAD test was replicated three times and each antenna used was from a different fly.

Coupled GC-EAD analysis of headspace volatiles collected from live either male or female adult R. batava showed that antennae of both sexes responded strongly to the single compound in the headspace of males (Figure 3). The antennae of males did not respond to any compound in the female headspace. 

GC-MS analyses of the volatiles collected from *R. batava* males demonstrated that the EAD active compound (retention index (RI) 1882 on Stabil-Wax column) produced a weak molecular ion at *m*/*z* 128. Significant ions included *m*/*z* (%) 42 (90), 55 (60), 71 (80) and 99 (100). The RI and mass spectrum of this compound matched those of the synthetic standard of δ-heptalactone.

### 3.2. EAD Active Enantiomer of δ-Heptalactone

**Figure 4:**
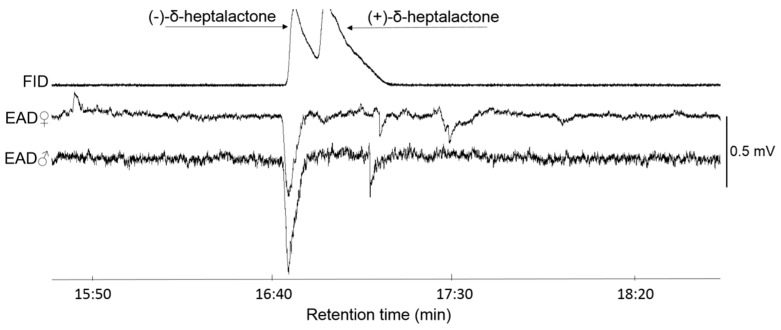
GC-EAD response of male and female *R. batava* to synthetic enantiomers of δ-heptalactone. Enantiomeric separation of δ-heptalactone was conducted using an Rt^®^-bDEXsm column (30 m × 0.25 mm × 0.25 µm, Restek Corporation); each EAD test was replicated three times and each antenna used was from a different fly.

Couplet GC-EAD analysis of synthetic enantiomers of δ-heptalactone revealed a strong response of *R. batava* fruit flies of both sexes to the single enantiomer only. No response to (+)-δ-heptalactone was registered and high sensitivity to (-)-δ-heptalactone was recorded (Figure 4).

### 3.3. Active Enantiomer of δ-Heptalactone in R. batava

**Figure 5:**
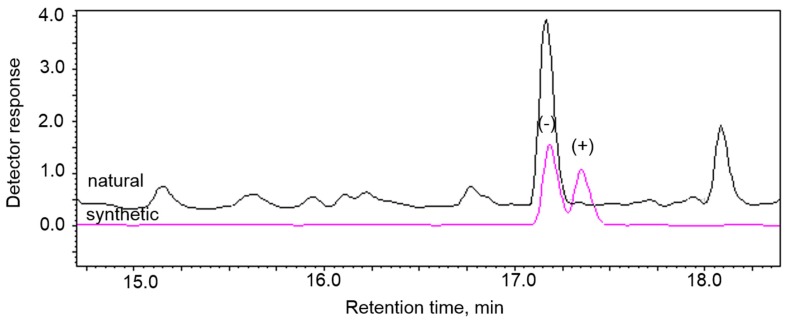
GC analyses of natural and synthetic enantiomers of δ-heptalactone on chiral Rt^®^-bDEXsm capillary column.

The enantiomeric composition of the insect-produced compound was determined to be (-)-δ-heptalactone by comparison of the retention time of natural δ-heptalactone with synthetic isomers on a chiral Rt^®^-bDEXsm capillary column (Figure 5).

### 3.4. Dose Response

**Figure 6:**
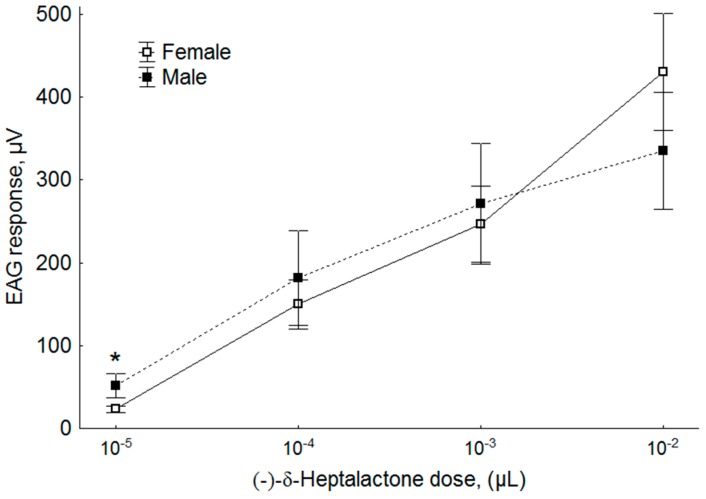
Electroantennogram (EAG) responses (mean amplitude ± standard error (SE), mV) of male and female *R. batava* to different doses (1 × 10^−5^ to 1× 10^−2^ µL) of synthetic (-)-δ-heptalactone. The asterisk denotes significant differences in EAG responses between sexes (Mann–Whitney U Test, Z = −2.08 *p* = 0.037); each EAD test was replicated five times and each antenna used was from a different fly.

The sensitivity of males and females to the lowest dose tested was different, with males being more sensitive than females (Mann-Whitney U test, Z = −2.08, *p* = 0.037). At higher doses, no differences in EAG responses between sexes was recorded (Mann–Whitney U test, Z = −0.3203, 0.2402 and 0.8807 at doses of 1 × 10^−4^, 1 × 10^−3^ and 1 × 10^−2^ µL, respectively, *p* > 0.05) (Figure 6).

### 3.5. Behavioural Test under Field Conditions

**Figure 7:**
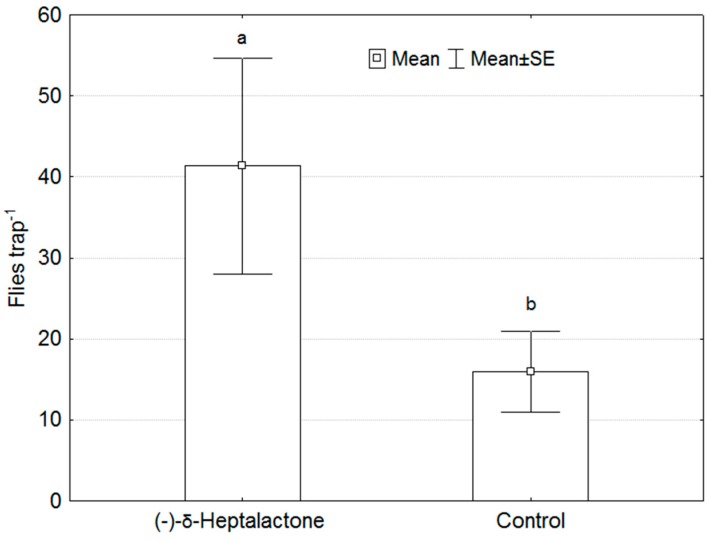
Field catches of *Rhagoletis batava* in McPhail traps. Different letters indicate statistically significant difference (general linear model, F = 7.013, *p* = 0.027); intervals mark SE; *n* = 3 for each type; trapping period lasted from 30 July to 20 August 2019.

Sex determination of the specimens caught by the traps revealed that both males and females of *R. batava* flies were captured. In traps loaded with (-)-δ-heptalactone, we caught 61 male and 63 females and the number of insects caught exceeded that of control traps by 2.5 times (Figure 7). 

Statistical data evaluation by GLM revealed that the treatment type (pheromone and control) significantly influenced the attraction of flies (F = 7.013, *p* = 0.027) whereas attraction between the sexes (male or female) was not significantly different (F = 0.061, *p* = 0.812). Twenty ± 6 (mean of specimens per trap ± standard error of mean) males and 21 ± 7 females were caught by the traps baited with (-)-δ-heptalactone and 7 ± 3 males and 8 ± 2 females we detected in the control traps. As the traps contained fruit flies of both sexes, we concluded that (-)-δ-heptalactone functions as an aggregation pheromone.

## 4. Discussion

In the majority of Tephritidae species, mating is mediated by a combination of chemical, acoustic and visual cues. Pheromones play a key role during the mate-searching phase and, in general, they are produced by males to attract females [15,23]. The results of our experiments showed that *R. batava* antennae from both sexes react to a single compound, δ-heptalactone, detected exclusively in the headspace emissions from males. No significant difference in EAG responses between sexes was recorded except testing the lowest 10^−5^ μg dose and males showed higher sensitivity than females. We assume that ability of males to sense lower dose of pheromone could allow them to detect single pheromone releasing males and to form groups of individuals for the purpose of mating, while females detect aggregations of the calling males perceiving higher concentrations of a pheromone. The responses of antennae to synthetic (-)- and (+)-enantiomers of δ-heptalactone revealed that only (-)-δ-heptalactone was EAD active while no response was registered to (+)-δ-heptalactone. Usually, receptors activated by optically active compounds show preferential of varying degrees to one of the enantiomers or respond at a similar level to both enantiomers [24,25,26,27]. Until date, no response to one enantiomer of an enantiomer pair has been reported in limited number of insect species [24,28,29].

**Figure 8:**
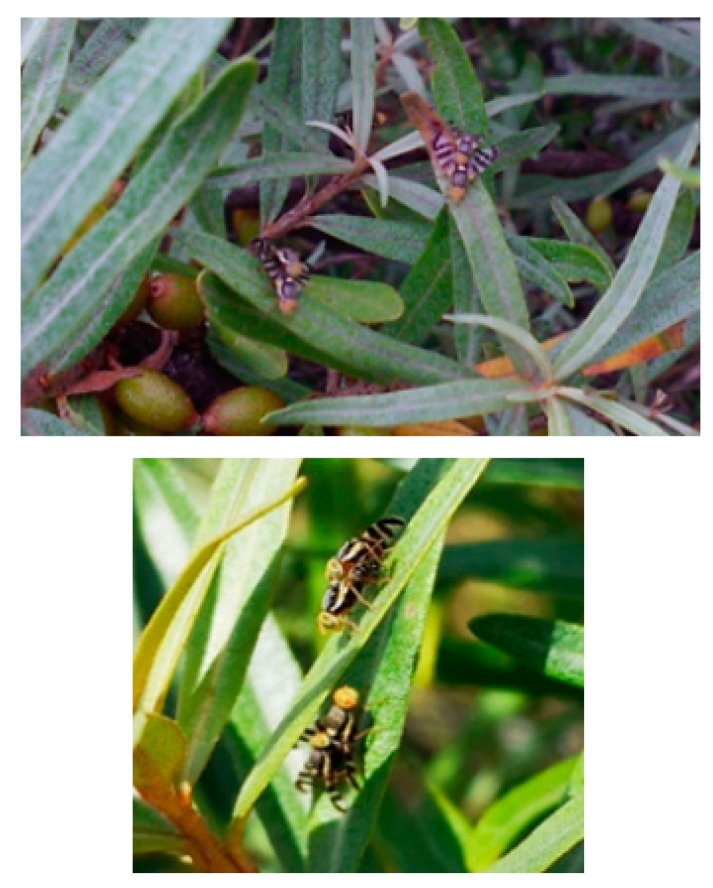
A few *R. batava* pairs in copula are commonly found near each other on adjacent leaves of a host plant *Hippophae rhamnoides*.

The attractiveness of the electrophysiologically active compound was also registered for both sexes under field conditions, revealing that the compound functions as a long-range attraction pheromone. Considering the definition given by Wyatt, “When male insects pheromone-call they often attract males as well as females; the pheromones concerned are called aggregation pheromones even if their original function was sex attraction” [30], we argue that the pheromone is attributed to the aggregation pheromone type. Many Tephritidae species form leks [31]; however, males of some *Rhagoletis* species (including *R. pomonella*, *R. rubicola* and *R. mendax*) individually seek out females on the fruit surface [32]. During our previous project studying the seasonal flight and mating dynamics of *R. batava* flies, 1847 pairs in copula were counted [21] and often a few mating pairs or a few individuals were observed at closer distance to each other rather than distributed evenly (Figure 8, personal report). Aluja and Birke defined a ‘‘lek’’ as an aggregation of at least three pheromone-calling males in a clearly defined area, usually from adjacent leaves of a host or non-host plant species [33]. Further studies are needed to collect more detailed information about the spatial and temporal characteristics of groups formed in response to male-released pheromones in the natural habitat of the species. 

Pheromone of *R batava* is a lactone formed by the cyclization of a hydroxyl ester [34]. To date, eight compounds bearing the lactone moiety have been reported in seven Tephritidae species, attributed to genera *Anastrepha*, *Bactrocera*, *Ceratitis* and *Rhagoletis* (Table 2). In all seven species, lactones were emitted exclusively by males (Table 2); hence, our data showing that (-)-δ-heptalactone is a male-specific compound follows the pattern observed in other Tephritidae species. The biological activity of lactones produced by *Anastrepha*, *Bactrocera*, *Ceratitis* and *Rhagoletis* males were tested using either just females or the sex of the specimens caught in the traps was not determined; hence, information is insufficient to determine whether males respond to the lactones as well.

To avoid toxic residues of insecticides in harvested sea buckthorn berries, biological or biotechnical control methods to decrease yield losses against *R. batava* flies are urgently required, while in organic production, biological control is only an option [35]. Different approaches could be considered including yellow sticky traps, insect sterilization and various biological antagonists, as well as semiochemical-based pest control means [36,37]. Our experimental field results showed that McPhail traps with a pheromone lure were over twice as efficient as trapping *R. batava* flies with control traps. Our previous work revealed a blend composed of nine EAD active yeast volatiles that attracted *R. batava* flies under laboratory conditions [38]. Further work is needed to increase lure efficiency by combining the pheromone with berry-associated yeast odors to develop a semiochemical-based trap for monitoring and control of *R. batava* flies in buckthorn orchards.

## 5. Conclusions

(-)-*δ*-heptalactone is emitted exclusively by males of *R. batava* flies. Only (-)-enantiomer elicited EAG responses in both sexes, whereas no response to the (+)-enantiomer was registered. Both males and females of *R. batava* were captured in traps baited with the active compound. Based on the mode of action, (-)-δ-heptalactone is an aggregation pheromone, with potential for application in pest management programs of *R. batava* flies. The aggregation type of a pheromone is first reported here within the genus *Rhagoletis*.

## Figures and Tables

**Table 1:** Yield and purity of δ-heptalactones.

Catalyst	Product	Yield	ee ^1^	CP ^2^
(1*R*,2*S*)-(+)-*N*,*N*-dibutylnorephidrine	(R)-(+)-**3**	35% over 2 steps	84%	97%
(1*S*,2*R*)-(−)-*N*,*N*-dibutylnorephidrine	(S)-(−)-**3**	32% over 2 steps	86%	96%

^1^ Enantiomeric excess; ^2^ CP, chemical purity, indicates percentage of both enantiomers.

**Table 2:** Lactones produced by Tephritidae species.

Compound Name	CAS No. ^1^	Species	Emitter	Reference
Lavender lactone	1073-11-6	*Anastrepha fraterculus*	M	[39]
Suspensolide	111351-08-7	*Anastrepha fraterculus*	M	[39,40,41]
		*Anastrepha ludens*	M	[42]
		*Anastrepha suspensa*	M	[42,43,44]
		*Anastrepha* sp.	M	[45]
S,S-Anastrephin	77670-94-1	*Anastrepha fraterculus*	M	[39,40,41]
		*Anastrepha ludens*	M	[42,46,47,48]
		*Anastrepha suspensa*	M	[42,43,44,46,48]
		*Anastrepha* sp.	M	[45]
S,S-Epianastrephin	77670-93-0	*Anastrepha fraterculus*	M	[39,40,41]
		*Anastrepha ludens*	M	[42,46,47]
		*Anastrepha suspensa*	M	[42,43,44,46,48]
		*Anastrepha* sp.	M	[45]
(E)-5-(3,6-heptadienyl)-dihydro-2(3H)-furanone	81693-14-3	*Bactrocera cucurbitae*	M	[49]
Dihydro-3-methylfuran-2(3H)-one	1679-47-6	*Ceratitis capitata*	M	[50]
*δ*-Hexalactone	823-22-3	*Rhagoletis completa*	M	[15]
*δ*-Heptalactone	3301-90-4	*Rhagoletis completa*	M	[15]

^1^ Chemical Abstract Service Number; M, male.
